# Airway management may influence postoperative ventilation need in preterm infants after laser eye treatment

**DOI:** 10.1038/s41390-024-03356-4

**Published:** 2024-06-22

**Authors:** Sarolta H. Trinh, Gyula Tövisházi, Lóránt K. Kátai, Luca L. Bogner, Erika Maka, Vera Balog, Miklós Szabó, Attila J. Szabó, János Gál, Ágnes Jermendy, Balázs Hauser

**Affiliations:** 1 Department of Neonatology, Paediatric Centre, MTA Center of Excellence, Semmelweis University, Budapest, Hungary; 2 Department of Anaesthesiology and Intensive Therapy, Semmelweis University, Budapest, Hungary; 3 Institute of Anaesthesiology and Perioperative Care, Semmelweis University, Budapest, Hungary; 4 Department of Ophthalmology, Semmelweis University, Budapest, Hungary

## Abstract

**Background:**

Retinopathy of prematurity is treated with laser photocoagulation under general anaesthesia with intubation using endotracheal tube (ETT), which carries a risk for postoperative mechanical ventilation (MV). Laryngeal mask airway (LMA) may provide a safe alternative. We assessed the need for postoperative MV in preterm infants who received LMA versus ETT.

**Methods:**

In this single-centre, retrospective cohort study, preterm infants who underwent laser photocoagulation between 2014–2021 were enroled. For airway management, patients received either LMA (*n* = 224) or ETT (*n* = 47). The outcome was the rate of postoperative MV.

**Results:**

Patients’ age were 37 [35;39] weeks of postmenstrual age, median bodyweight of Group LMA was higher than Group ETT’s (2110 [1800;2780] g versus 1350 [1230;1610] g, respectively, *p* < 0.0001). After laser photocoagulation, 8% of Group LMA and 74% of Group ETT left the operating theatre requiring MV. Multiple logistic regression revealed that the use of LMA and every 100 g increase in bodyweight significantly decreased the odds of mechanical ventilation (OR 0.21 [95% CI 0.07–0.60], and 0.73 [95% CI 0.63–0.84], respectively). Propensity score matching confirmed that LMA decreased the odds of postoperative MV (OR 0.30 [95% CI 0.11–0.70]).

**Conclusion:**

The use of LMA is associated with a reduced need for postoperative MV.

**Impact:**

- Using laryngeal mask airway instead of endotracheal tube for airway management in preterm infants undergoing general anaesthesia for laser photocoagulation for treating retinopathy of prematurity could significantly decrease the postoperative need for mechanical ventilation. According to our current understanding, this has been the largest study investigating the effect of laryngeal mask airway during general anaesthesia in preterm infants. Our study suggests that the use of laryngeal mask airway is a viable alternative to intubation in the vulnerable population of preterm infants in need of laser treatment.

## Introduction

Very low birth weight (VLBW) infants are at a high risk of developing typical comorbidities that are associated with preterm birth, including retinopathy of prematurity (ROP), a vasculo-proliferative disease of the eye. ROP has a high incidence in infants whose gestational age is below 30 weeks and whose birth weight is less than 1300g.^1^ Severe forms of ROP, stage 3–4, require ophthalmic intervention, which can be laser photocoagulation, intravitreal anti-vascular endothelial factor injection or progressive stage 4 ROP may require vitreoretinal surgery. For these therapies, general anaesthesia is the safest anaesthetic method providing patients with painlessness and complete immobility for the ophthalmologist to facilitate the most precise intervention.

Anaesthesia of this patient population is challenging, given their immature physiology and comorbidities, especially bronchopulmonary dysplasia (BPD), which is associated with extended periods of previous ventilatory support.^2^ Moreover, VLBW infants have numerous complicating factors, comorbidities,^3,4^ all increasing the risk of anaesthetic care. There is an increasing initiative to take VLBW infants to the operating room for laser photocoagulation to provide the safest possible care. The endotracheal tube (ETT) is the gold standard of airway management in general anaesthesia, ensuring a safe airway and protecting against aspiration. In contrast, there is growing evidence that laryngeal mask airway (LMA) provides a safe alternative with an easier insertion and less airway irritation. Furthermore, the use of LMAs avoids laryngoscopy and tracheal intubation, which can lead to significant intracranial, intraocular, and systemic blood pressure elevation.^5,6^ Finally, by applying LMAs it is possible to avoid the use of neuromuscular blocking agents, thus decrease the pharmacological risk in premature infants.^2^ Therefore, several studies point out that the use of LMA instead of ETT results in fewer adverse events in infants and premature infants and could hence be a good or even better alternative to ETT.^7–13^

To date, no study has compared the effects of LMA and ETT on the need for postoperative ventilatory support in VLBW infants. Therefore, we designed a retrospective cohort study with the hypothesis that the need for postoperative invasive ventilation is lower in infants who received an LMA, than in those who received ETT for airway management during general anaesthesia for laser treatment of ROP.

## Materials and methods

This retrospective cohort study was conducted at the Paediatric Centre of Semmelweis University, Budapest, Hungary, after the approval was granted by the Institutional Ethics Committee (license number SE RKEB 33/2022). As this was a retrospective study, the requirement for written informed consent was waived by the Institutional Ethics Committee. We enroled VLBW infants who underwent anaesthesia for laser photocoagulation between August 2014 and December 2021. Data analysis and statistical plan were defined before accessing the research data and submitted to the Institutional Review Board.

### Study population

All consecutive VLBW infants who underwent general anaesthesia for laser photocoagulation in the operating room in the study period were eligible to participate in this study. We evaluated clinical data which was abstracted from the electronic health record. Infants whose electronic health records were incomplete or who arrived at the operating room with mechanical ventilatory support were excluded from this study.

### Study protocol and clinical care of patients

Our institution is a regional centre for neonatal laser photocoagulation treatment, all outborn infants were referred from other neonatal intensive care units on the day before the scheduled treatment. Before the laser photocoagulation, patients had been enteral fasting for 4–6 h for breast milk and/or formula and 1 or 2 h for clear fluids, according to the current European recommendations, respectively.^14,15^ In our institute, neonatal laser photocoagulation is routinely performed under general anaesthesia, with the following premedication: acetaminophen 15 mg/kg once per rectum and tropicamide and phenylephrine eyedrops four times over 1 h before laser photocoagulation. During the procedure standard anaesthetic monitoring was performed in the operating theatre using a pulse oximeter, electrocardiogram, non-invasive blood pressure monitor, thermometer and capnograph. Anaesthesia was induced by sevoflurane inhalation via facemask, aiming to achieve high MAC values and minimise opiate use. When the appropriate depth of anaesthesia was achieved the type of airway device was selected at the discretion of the attending paediatric anaesthesiologist. Patients received either LMA (Aura40™, Ambu®, Ballerup, Denmark or LMA® Classic™ Airway Teleflex®, Wayne, Pennsylvania, size 1) or uncuffed endotracheal tube (size 3 or 3.5). Decision to intubate or use LMA was based on several factors, including body weight and actual clinical status of the infant, and previous personal experience of the paediatric anaesthesiologist. Neuromuscular blocking agents (succinylcholine, atracurium and mivacurium) were not used for LMA insertion and there was an initiative to minimise the use for ETT insertion as well, but it was left at the discretion of the attending paediatric anaesthesiologist. During the retrospective chart review, we placed patients to LMA and ETT groups, based on the primary method intended for airway management by the anaesthesiologist. Infants were maintained on a mixture of oxygen, air and sevoflurane with pressure support or pressure-controlled ventilation used as needed. At the end of the laser photocoagulation procedure, LMA was removed, and extubation was attempted in all cases in patients with ETT. If extubation was unsuccessful or not attempted at the end of surgery, patients remained on invasive ventilation. Subsequently, all patients were admitted to the neonatal intensive care unit (NICU) for postoperative observation. As for the physicians providing care, all paediatric anaesthesiologists were senior specialists in this unit with more than 10 years of experience in neonatal anaesthesia and the same paediatric ophthalmologist performed the LPC therapy.

### Outcome measures

The primary outcome was the rate of postoperative mechanical ventilation (MV) via ETT after laser photocoagulation in the two groups. The following covariates were collected anonymized: date of birth, date of the laser photocoagulation, gestational age at birth, birth weight, previous days on the MV cumulatively, need for postoperative MV, length of invasive or non-invasive ventilatory support and type if needed, days spent at the NICU after laser photocoagulation, perioperative complications, comorbidities. For comorbidities we collected data on necrotising enterocolitis, periventricular leukomalacia or intraventricular haemorrhage grade III/IV., and sepsis. We assigned one point for each comorbidity on a three-point scale. From the anaesthesia reports the following data were extracted and anonymized: postconceptual age at the time of laser photocoagulation, need for ventilatory support before laser photocoagulation, length of general anaesthesia and length of laser photocoagulation, airway management, intraprocedural complication. Many of these covariates were selected as possible predictors of the need for postoperative mechanical ventilation.

### Statistical analysis

Descriptive statistics are expressed as median with interquartile ranges [IQR] for continuous variables or as a number with percentage. Cases where key covariates were missing were excluded from the study. Mann–Whitney U test and Chi-square test were used to compare clinical characteristics and outcomes of the two groups. Significance was determined at *p* < 0.05. Due to retrospective study design, we performed a compound statistical analysis to minimise selection bias and clinical differences between the study groups. We followed a two-step approach to detect a possible relationship between airway management (LMA or ETT) and the need for MV. First, in univariate analyses, unadjusted models were built to study the relationships between airway management, and other clinically relevant parameters as predictors and postoperative MV as outcome. On the basis of a priori clinical knowledge the following variables were selected: gestational age, mechanical ventilation, bodyweight at the time of laser photocoagulation, length of general anaesthesia, airway management and major comorbidities (including necrotising enterocolitis, periventricular leukomalacia or intraventricular haemorrhage gr. III/IV., and sepsis). One point was assigned to each comorbidity on a three-step scale. Next, a multivariable logistic regression model was developed to ascertain the effect of airway management on the likelihood that patients would need postoperative MV, adjusting for the other clinically relevant parameters. Results are presented as unadjusted and adjusted odds ratios with 95% confidence intervals (CI). Finally, a propensity score adjustment was applied to improve the association between airway management and outcome and to address the difference in the number of patients in the two groups. Matching was implemented with the nearest 1:1 neighbour propensity score matching without replacement. In order to reduce the bias due to residual imbalance and make the effect estimate doubly robust, we have included the covariates in the outcome model after matching, and the odds ratio and its 95% CI were estimated with ordinary nonparametric bootstrap method based on 499 bootstrap replicates. The data were analysed using GraphPad Prism, version 9 (GraphPad Software, San Diego, CA) and R Statistical software 4.1.3 (R Foundation for Statistical Computing, Vienna, Austria).

## Results

**Fig. 1:**
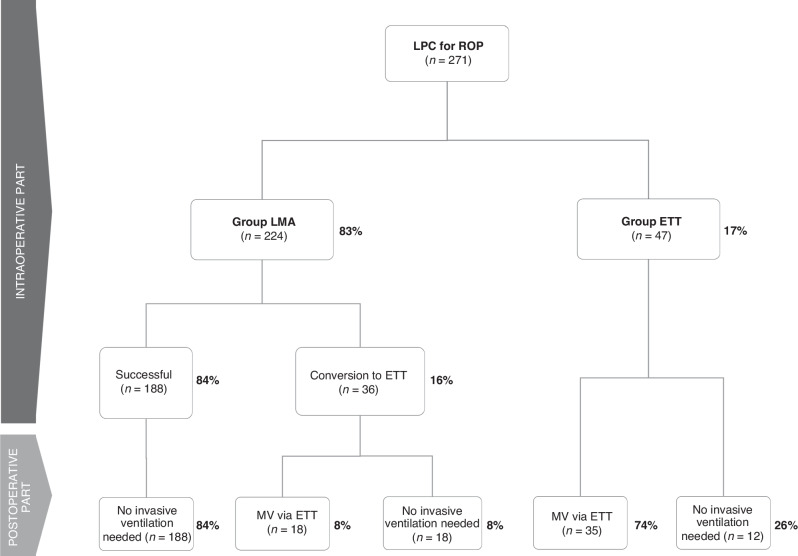
Flowchart of the infants included in the study and their outcomes. ETT endotracheal tube, LMA laryngeal mask airway, LPC laser photocoagulation, MV mechanical ventilation, ROP retinopathy of prematurity.

Of the 296 infants undergoing general anaesthesia in the operating room for laser photocoagulation treatment for ROP between August 2014 and December 2021 we included 271 VLBW infants as 16 infants were excluded due to arriving on MV to the OR and 9 patients were excluded for missing data. Patients were divided into two groups based on the decision of the attending anaesthesiologist on intended initial airway management: the LMA group (*n* = 224) and the ETT group (*n* = 47). In the LMA group we distinguished two clinical scenarios, yielding two subgroups, as not every patient tolerated LMA well, and 16% (36/224) of these infants required conversion to ETT (successful LMA and conversion to ETT subgroup, respectively, see Fig. 1). The clinical parameters of the groups are shown in Table 1. Infants in the ETT group were younger, had lower body weight and required longer laser treatment. Supplementary Table 1. shows the severity of ROP, affected zones, and characteristics of the laser treatment. Supplementary Table 2. demonstrates that the rate of intraoperative complications was similar in the two groups.

**Table 1:** Clinical characteristics of the study population.

Clinical data						
Variables	Total population (*n* = 271)	Group LMA (*n* = 224)	Successful LMA (*n* = 188)	Conversion to ETT (*n* = 36)	Group ETT (*n* = 47)	*p* value
Gestational age at birth (weeks)	26 [25, 28]	26 [25, 28]	26 [25, 28]	25 [24, 26]	25 [24, 26]	<0.001
Birth weight (g)	788 [650, 985]	810 [700, 990]	840 [700, 1080]	750 [630, 865]	640 [538, 850]	<0.001
Age at laser photocoagulation (weeks)	37 [35, 39]	37 [35, 39]	38 [35, 40]	36 [34, 37]	35 [33, 36]	<0.001
Body weight at laser photocoagulation (g)	2018 [1625, 2660]	2110 [1800, 2780]	2250 [1845, 2945]	1800 [1585, 2160]	1375 [1235, 1600]	<0.001
MV before LPC (days)	14 [4, 27]	11 [3, 25]	10 [2, 22]	20 [8, 35]	29 [16, 40]	<0.001
Length of general anaesthesia for laser photocoagulation (min)	95 [75, 125]	86 [70, 110]	85 [65, 101]	108 [95, 140]	138 [105, 160]	<0.001
Length of laser photocoagulation (min)	60 [45, 80]	57 [42, 75]	55 [40, 71]	72 [52, 95]	90 [70, 115]	<0.001
Extubation rate at the end of laser photocoagulation	80% (218)	92% (206)	100% (188)	50% (18)	27% (12)	<0.001

Data shown as median [IQR]. See text for details.

*P* values represent comparisons between Group LMA and Group ETT.

*ETT* endotracheal tube, *LMA* laryngeal mask airway, *MV* mechanical ventilation.

### Outcomes

After laser photocoagulation, the anaesthesiologist removed the LMA or attempted to extubate all patients on ETT. If the extubation was unsuccessful and patients required continuous invasive ventilatory support, they were moved to the MV outcome group, otherwise no postoperative invasive ventilation was required. Postoperative MV was required by 74% (35/47) of the patients in the ETT group compared to 8% (18/224) in the LMA group (*p* < 0.001). Furthermore, infants in the ETT group needed observation and ventilatory support in the NICU for a longer time. Data of clinical findings after laser photocoagulation are shown in Table 2. Additional postoperative complications are shown in Supplementary Table 2. It is noteworthy, that postoperative complication rate was higher in Group ETT, but due to low absolute numbers, the clinical significance of this finding is unknown.

**Table 2:** Post- laser photocoagulation clinical characteristics.

Post- laser photocoagulation clinical characteristics			
Variables	Group LMA (*n* = 224)	Group ETT (*n* = 47)	*p* value
Post- laser photocoagulation observational time at the NICU (days)	2 [2, 3]	3.5 [2, 18]	<0.001
Distribution of ventilatory support			
No ventilatory support	188 (84%)	7 (15%)	<0.001
Non-invasive (HFNC, CPAP, BiPAP)	18 (8%)	5 (11%)	
Invasive	18 (8%)	35 (74%)	
Duration of post- laser photocoagulation MV (days)	0 [0, 0.4]	2 [1, 5]	<0.001

Data are shown as median [IQR] or number (%).

*BiPAP* bilevel positive airway pressure, *CPAP* continuous positive airway pressure, *ETT* endotracheal tube, *HFNC* high flow nasal cannula, *LMA* laryngeal mask airway, *NICU* neonatal intensive care unit, *MV* mechanical ventilation.

A two-step approach was used to establish the relationship between airway management (LMA or ETT) and the need for MV after surgery. First, we performed a logistic regression analysis, second, a propensity score matching.

### Logistic regression analysis

In the univariate logistic regression analysis all variables were found to be significant in influencing the outcome. The unadjusted models are shown in Table 3. After adjusting for confounding factors, the analysis revealed that using an LMA for airway management decreases the odds of remaining on the ventilator postoperatively to one-fifth. In addition, longer previous mechanical ventilation, longer anaesthesia, and a higher number of major comorbidities increase the likelihood of postoperative MV, whereas a body weight of 100 g more at the time of laser photocoagulation decreases the odds by 27%. Interestingly, the age at the time of laser photocoagulation was not a significant predictor of the postoperative MV outcome.

**Table 3:** Results of logistic regression analysis.

Factors influencing the need for post-laser photocoagulation ventilatory support				
Variables	Unadjusted odds ratios		Adjusted odds ratios	
	OR	95% CI	aOR	95% CI
Airway management (LMA vs ETT as reference)	0.03	0.01–0.07	**0.21**	**0.07–0.60**
Gestational age (weeks)	0.62	0.49–0.76	0.76	0.50–1.11
Previous MV (days)	1.07	1.05–1.10	**1.07**	**1.03–1.11**
Bodyweight at laser photocoagulation (in 100 g)	0.72	0.65–0.80	**0.73**	**0.63–0.84**
Length of anaesthesia (min)	1.03	1.02–1.04	**1.02**	**1.01–1.03**
Major comorbidity	2.65	1.90–3.87	**2.02**	**1.16–3.68**

Adjusted odds ratios of factors with statistical significance are set in bold.

Major comorbidities: Necrotising enterocolitis, periventricular leukomalacia or intraventricular haemorrhage gr. III/IV., sepsis. One point was given for each comorbidity on a three-step scale.

*aOR* adjusted odds ratio, *CI* confidence interval, *ETT* endotracheal tube, *LMA* laryngeal mask airway, *MV* mechanical ventilation, *OR* odds ratio.

### Propensity adjusted analysis

To further address confounders by indication (LMA or ETT) and to improve the causal association with the outcome, we performed a propensity score analysis. On the basis of a priori clinical knowledge, we used the aforementioned clinical factors for a 1:1 propensity score matching for all ETT patients (*n* = 47), creating a subgroup of propensity matched LMA group (*n* = 47). The clinical characteristics of these patients are shown in Table 4. When calculating the odds for the need of postoperative MV, the bootstrap method yielded an odds ratio of 0.30, with 95% CI: 0.11–0.70 for patients with LMA compared to patients with ETT. These results are similar to those of the logistic regression analysis.

**Table 4:** Propensity score matching.

1:1 Propensity score matching				
	Before matching		After matching	
Variables	Group LMA (*n* = 217)^a^	Group ETT (*n* = 47)	Propensity matched Group LMA (*n* = 47)	Group ETT (*n* = 47)
Birth weight (g)	890 (310)	680 (190)	730 (230)	680 (190)
Gestational age (weeks)	26 (2.3)	25 (1)	26 (2)	25 (1)
Previous MV (days)	15 (15)	30 (19)	25 (14)	30 (19)
Body weight at laser photocoagulation (g)	2360 (790)	1460 (390)	1640 (200)	1460 (390)
Major comorbidities	0.69 (0.85)	1.36 (0.94)	1.23 (0.94)	1.36 (0.94)
Odds ratio for postoperative MV (LMA vs ETT): 0.30 (95% CI: 0.11–0.70)				

Major comorbidities: One point for each: Necrotising enterocolitis, periventricular leukomalacia or intraventricular haemorrhage gr. III/IV., sepsis.

Data are shown as mean (SD).

*ETT* endotracheal tube, *LMA* laryngeal mask airway, *MV* mechanical ventilation.

^a^After removing patients with missing data regarding the 5 variables taken into account in the propensity matching.

## Discussion

Our results suggest that the use of LMA compared to ETT is independently associated with a reduced need for postoperative invasive ventilation in VLBW infants with ROP undergoing laser photocoagulation. Our data show that the odds of postoperative mechanical ventilatory support are at least three times lower (based on propensity matching) with LMA than with ETT.

With positive pressure ventilation, supraglottic devices will always have a potential risk of aspiration of gastric content; thus, ETT is a safer option for securing the airway. However, there are several papers proving that LMA is a safe alternative in several clinical scenarios, and also reduces perioperative complications. In a former study comparing LMA, cuffed ETT and uncuffed ETT, it was reported that the use of LMA reduced the incidence of postoperative laryngospasm and coughing in infants aged between 1 to 24 months undergoing lower abdominal surgery.^10^ Drake-Brockman and colleagues found in a randomised control trial that the incidence of perioperative respiratory adverse events were three times higher in patients receiving ETT than in patients receiving LMA in an infant population under 1 year of age who underwent elective general anaesthesia.^8^ Another study comparing LMA and ETT in 70 neonates demonstrated that extubation was longer in patients who received ETT and had more postoperative respiratory adverse events.^11^ Previously, a small case series study suggested that LMA airway management was feasible in patients who underwent ROP treatment.^12^ Besides our results being consistent with the above-mentioned findings on LMA, our study also suggests that LMA may be a safe alternative to ETT in VLBW infants, providing safer care options for an especially vulnerable patient population. It is conceivable that LMA is less airway irritative and less likely to reactivate BPD, a common comorbidity in VLBW infants, thus reducing the risk for postoperative mechanical ventilatory support.

Previous studies have described the patterns of reintubation and its relationship to BPD in VLBW cohorts. In a cohort of infants with birth weight ≤1250 g, 47% were reintubated during hospitalisation, one-third of which for non-respiratory reasons, 5% for elective procedures.^16^ The same group has reported that time to reintubation independently modulated the odds of the combined outcome of death/BPD.^17^ Furthermore, others have found that infants reintubated within 7 days after extubation were significantly more likely to die or require prolonged respiratory support and hospitalisation.^18^ All these findings emphasize the notion that it is best to avoid invasive mechanical ventilation in VLBW infants who were once successfully extubated before. A ROP intervention related postoperative mechanical ventilation is not only a risk factor for further complications in an already compromised patient population, but also a financial burden for healthcare institutions as it does not only require a higher level of care, but also prolongs the length of stay of these infants in the NICU.

Further physiological considerations also support the use of LMA. Disma and colleagues stated that difficult intubation occurred in 5.8% of neonatal or infant anaesthesia, which was accompanied with a longer desaturation or bradycardia in a large amount of the cases.^19^ Moreover, a review suggested that hypoxia, hypotension and anaemia were the dreaded triad that increased morbidity and mortality in neonates undergoing anaesthesia.^20^ A recent review study pointed out that lately there is a shift from general anaesthesia towards other types of anaesthesia in preterm infants undergoing laser photocoagulation.^21^ The main reason for this phenomenon was to decrease adverse events associated with extubation postoperatively. These findings suggest that the use of LMA instead of ETT can increase patient safety by reducing the chance of possible adverse events associated with ETT and eliminating the use of muscle relaxants for ETT insertion in VLBW infants.

Although our data show advantages of LMAs, the device has some limitations and disadvantages.

Firstly, according to the manufacturer, the lowest limit of use of LMA is a body weight of 2000 g. In our experience, LMA can be used successfully even in patients weighing less than 1500 g. However, if the patient was too small, an anatomical mismatch might cause leakage, and the LMA might not seal properly, although we followed the instructions of the manufacturers, and LMA cuff pressure was checked using manometry. In these cases, when a leakage occurred and could not be solved in any other way, it led to conversion to ETT. Another possible issue is the problem of dead space due to the discrepancy between body weight of the infant and the size of the LMA, which can potentially lead to hypercapnia. Secondly, respiratory adverse events such as desaturation or hypercapnia are common in neonates.^22^ LMA, being a less invasive airway device, can be dislocated easily from its correct position, even with a slight touch by the ophthalmologist, resulting in respiratory compromise. While the anaesthesiologist is performing an intervention, ophthalmologic treatment has to be stopped due to the small body size of the patients and the fact that both the ophthalmologic intervention and the airway management take place at the head. Thus, any LMA dislocation can lengthen the entire procedure. It is important to point out that LMA airway management is not suitable for standard thoracic or abdominal surgeries in premature neonates; however, it may be considered in other minor surgical interventions besides laser photocoagulation, for example in inguinal hernia repair.

Our study has several strengths and limitations. According to our current understanding, this has been the largest study investigating the effect of LMA during general anaesthesia in VLBW infants. During the entire 7.5-year study period laser photocoagulations were performed by a single paediatric ophthalmologist, and anaesthesia was provided by the same team consisting of senior paediatric anaesthesiologists using uniform equipment and protocol for general anaesthesia. Due to the long study period, we could enrol a significant number of patients, which granted us the possibility to perform different and complex statistical analyses, all of which yielded very similar results.

Naturally, there are limitations to this study that should be noted. As an observational retrospective study, randomisation of patients was impossible. Airway management as a key determining factor on the outcome was entirely left to the discretion of the attending anaesthesiologist. Therefore, the choice of airway management was determined by whether the infant had been previously intubated and was dependent on mechanical ventilation, whether the infant had received any type of respiratory support (HFNC, NIV) or the body weight of the infant at the time of the laser photocoagulation. This can result in a selection bias, which we attempted to overcome by using complex statistical methods, multiple regression analysis and propensity score matching, and both methods yielded similar results. A further limitation may be that we did not collect and compared data other than respiratory ones, however the immediate benefit and safety of use of LMA instead of ETT expected in respiratory outcomes. Of note, we could not control for BPD as a comorbidity, as the majority of our patient were less than 36 weeks old at the time of laser photocoagulation, hence the BPD diagnosis could not be established at that point. However, we used the cumulative days of mechanical ventilation as a proxy measure, which predicts BPD and home oxygen need.^23^ Finally, we acknowledge that generalisability can possibly be a limitation of our study, as in some centres infants are not admitted to the operating theatre for laser photocoagulation treatment under general anaesthesia. However, our results may be relevant for the anaesthesia of other minor procedures in preterm infants.

## Conclusions

Providing general anaesthesia to preterm infants can be a demanding task for the paediatric anaesthesiologist as these patients could be at a higher risk for perioperative complications such as need for postoperative ventilation due to their comorbidities and immature physiology. In our retrospective cohort study, we found that the use of LMA during general anaesthesia in preterm infants undergoing laser photocoagulation for the treatment of ROP reduces the odds of postoperative mechanical ventilation compared to ETT. We suggest that the use of LMA during anaesthesia is a viable alternative for infants requiring ROP laser treatment. However, further research is warranted to study which subgroups of premature infants may benefit the most from LMA management during general anaesthesia.

## Supplementary information

Supplementary Information

## Data Availability

The datasets generated and analysed during the current study are available from the corresponding author on reasonable request.
