# Supplementary material for: Airway management may influence postoperative ventilation need in preterm infants after laser eye treatment
Source: Pediatr Res. 2024 Jun 22;97(1):341–7. doi: 10.1038/s41390-024-03356-4 (PMC11798834; doi:10.1038/s41390-024-03356-4)
Supplement: Supplementary file 1 — Supplementary Information [file 41390_2024_3356_MOESM1_ESM.pdf]

Supplemental File

Supplemental Table 1. The severity of ROP, affected zones and characteristics of the laser treatment.

	**Total population** (n=271)	**Group LMA** (n= 224)	**Group ETT** (n= 47)	**P values**
**ROP stages**				
**Stage 1**	0	0	0	
**Stage 2**	1	1	0	
**Stage 3**	143	199	44	
**Stage 4a**	2	2	0	
**Missing data**	24	21	3	
**Affected zones**				
**Zone I**	12 (4%)	9	3	
**Zone I&II**	24 (9%)	6	16	
**Zone II**	204 (75%)	179	25	
**Zone II&III**	9 (3%)	9	0	
**Zone III**	1 (0%)	1	0	
**Missing data**	23 (8%)	20	3	
**Laser spots right eye**	2924 [1949, 4237]	2659 [1716, 3855]	4237 [3156, 5693]	<0.0001
**Laser spots left eye**	2986 [1983, 4283]	2699 [1777, 3657]	4348 [3289, 5582]	<0.0001

Data are shown as absolute numbers (with percentages) or medians with interquartile ranges.
P values represent Mann-Whitney's test results, comparisons are made between Group LMA and Group ETT.
Statistical test were not possible to execute on ROP stages and zones affected due to 0 values in subgroups.
Abbreviations: ETT, endotracheal tube; LMA, laryngeal mask airway; NEC, necrotising enterocolitis.

Type I ROP is indication for treatment which can be any of the following: [1] Any stage ROP in zone I with plus disease [2] Stage 3 ROP in zone I without plus disease [3] Stage 2 or 3 ROP in zone II with plus disease

Supplemental Table 2. Intraoperative and postoperative complications.

	**Group LMA** (n= 224)	**Group ETT** (n=47)	p value
**Intraoperative complications**	7 (3.1%)	3 (6.4%)	0.385
**Postoperative complications**	14 (6.3%)	11 (23%)	**0.001**
Vital instability	10	3	
Sepsis	3	5	
Ileus/ NEC	1	2	
Pneumonia	0	1	

Intraoperative complications include desaturation, bronchospasm, apnea, bradycardia, or hypotension.
Postoperative complications occurred within 24 hours of the laser treatment.
Data are shown as absolute numbers.
P values represent Fischer’s exact test results.
Abbreviations: ETT, endotracheal tube; LMA, laryngeal mask airway; NEC, necrotising enterocolitis.
